# Does the Fc Region Have a Role in the Ocular Half-life After Intravitreal Injection?

**DOI:** 10.1167/iovs.61.5.20

**Published:** 2020-05-14

**Authors:** Antonello Caruso, Norman A. Mazer

**Affiliations:** ^1^ Roche Pharma Research and Early Development, Pharmaceutical Sciences, Roche Innovation Center Basel, F. Hoffmann-La Roche AG., Basel, Switzerland. E-mail: antonello.caruso@roche.com.

We have read with interest the article by Joo et al.^1^ entitled “Role of the Fc region in the vitreous half-life of anti-VEGF drugs,” which concludes that the fragment crystallizable (Fc) region is a determinant of ocular pharmacokinetics (PK) following intravitreal injection. In this correspondence, we show that the experimental data presented in the article suggests the contrary.

Joo et al.^1^ tested two molecules, a conventional VEGF-Trap and a Fc region-deficient VEGF-Trap (Fcf VEGF-Trap), in a New Zealand white rabbit model of ocular PK. Based on their analysis of the vitreous and retina/choroid concentrations, the authors found longer half-lives for Fcf VEGF-Trap. In concluding that the presence of the Fc region accelerates ocular drug elimination, the authors ignore and contradict their own analysis of the aqueous humor PK, where the reported half-life of the Fc-containing molecule is nearly double that of the Fc-deficient version (78.89 and 43.02 hours, respectively).

Moreover, the half-life values estimated by Joo et al. are problematic for three distinct reasons. First, the vitreal concentrations shown in Figure 3 are remarkably comparable between molecules, their values superimposing at several time points. Yet inexplicably, the fitted lines and the associated half-lives differ markedly (103.99 and 145.02 hours for VEGF-Trap and Fcf VEGF-Trap, respectively). Second, for each study molecule, the half-life values show up to threefold differences among aqueous humor, vitreous humor, and retina/choroid. This finding is in contrast with multiple previous studies, which demonstrated experimentally^2^^,^^3^ and theoretically^4^ that antibody drug concentrations in ocular tissues decline with essentially the same terminal decay rate (“flip-flop” kinetics in the aqueous humor and retina/choroid). Third, in the case of the VEGF-Trap measured in retina/choroid, only 4 data points obtained up to 5 days post injection are available for analysis. This limits the reliability of any estimate derived from these data. The VEGF-Trap half-life values in the other tissues (103.99 and 78.89 hours, i.e. 4.3 and 3.3 days, in vitreous and aqueous humor, respectively) also indicate that a longer period of observation would be required for a credible estimate, namely 2 to 4 half-lives.^5^^,^^6^

To address these methodological issues, the concentration data in the table were re-analyzed. The half-life values of both molecules in each matrix were determined by fitting the terminal phase to an exponential function (noncompartmental analysis, Certara Phoenix software version 6.4), as shown below in the Figure.

**Table.** Estimated Half-Life Values for VEGF-Trap and Fcf VEGF-Trap in Rabbit Ocular Tissues.

Half-life	Vitreous	Aqueous	
(days) (CV)	Humor	Humor	Retina/Choroid
VEGF-Trap	5.1 (17%)	6.9 (16%)	–
Fcf VEGF-Trap	5.8 (23%)	7.0 (71%)	6.9 (12%)

CV, coefficient of variation.

**Figure.**
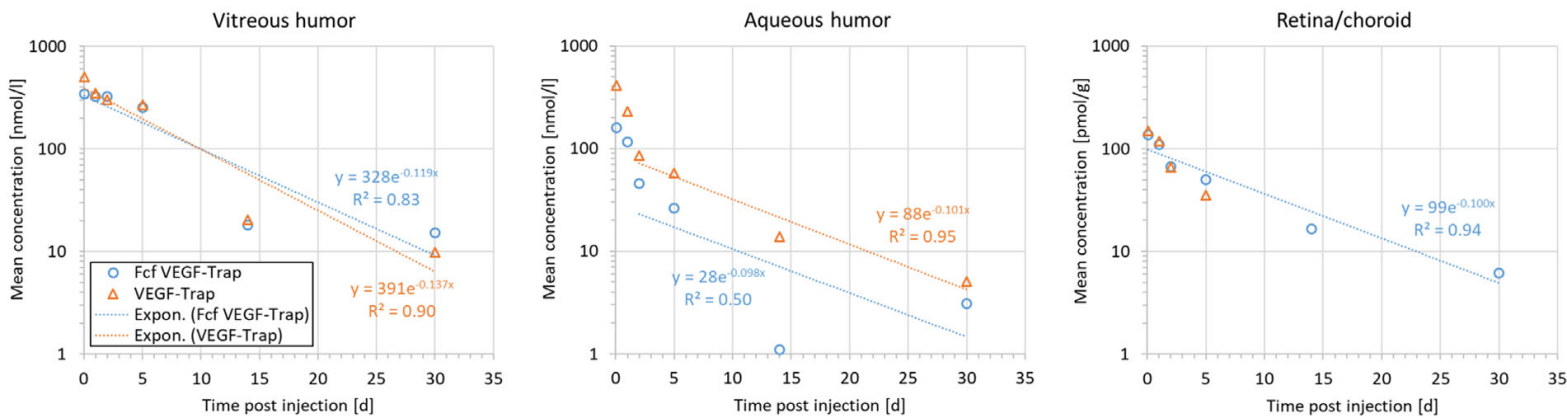
Semi-logarithmic plots of the concentration-time course for VEGF-Trap and Fcf VEGF-Trap in rabbit eyes. Symbols: experimental data by Joo et al.^1^ Lines: linear regression of the terminal elimination phase. The exponential function equation reports the estimated decay rate constant, from which the half-life value is calculated. Exclusion of day 14 concentrations, which may be considered outliers, does not lead to meaningfully different half-life estimates (not shown).

The resulting half-life estimates and uncertainties (coefficient of variation) are shown in the Table. A value for VEGF-Trap in the retina/choroid was not estimated due to the limited data. As expected, the ocular half-lives are comparable between tissues for both VEGF-Trap and Fcf VEGF-Trap. Comparing the results in the vitreous and aqueous humor, we find no meaningful difference between the study molecules.

In conclusion, our re-analysis of the concentration data presented by Joo et al.^1^ does not substantiate a difference in ocular elimination associated with the Fc region, consistent with what has been reported previously by Gadkar et al.^2^
